# Identifying the Role of GluN2A in Cerebral Ischemia

**DOI:** 10.3389/fnmol.2017.00012

**Published:** 2017-01-24

**Authors:** Yongjun Sun, Long Wang, Zibin Gao

**Affiliations:** ^1^Department of Pharmacy, Hebei University of Science and TechnologyShijiazhuang, China; ^2^Hebei Research Center of Pharmaceutical and Chemical Engineering, Hebei University of Science and TechnologyShijiazhuang, China; ^3^Department of Family and Consumer Sciences, California State UniversityLong Beach, CA, USA; ^4^State Key Laboratory Breeding Base—Hebei Province Key Laboratory of Molecular Chemistry for DrugShijiazhuang, China

**Keywords:** cerebral ischemia, GluN2A, triheteromeric NMDA receptor, expression, pharmacological properties

The activity of the NMDA receptor (NMDAR), which is a glutamate-gated ion channel, is a key factor influencing the neuronal damage following cerebral ischemia. NMDAR is composed of two essential GluN1 subunits and two regionally localized GluN2 or GluN3 subunits. GluN1, GluN2A, and GluN2B are the primary NMDAR subunits in the adult forebrain. Thus, the three major NMDAR subtypes in the adult forebrain are the GluN1/2A receptor, the GluN1/2B receptor, and the GluN1/2A/2B receptor. The functional properties of these NMDARs depend mainly on the GluN2 subunits, and many studies primarily classify NMDARs as GluN2A- and GluN2B-containing NMDARs. However, given the influence of the triheteromeric GluN1/2A/2B receptor, this misclassification might lead to a misunderstanding of the physiological and pathological roles of GluN2, especially the GluN2A subunit. Understanding the role of the GluN1/2A/2B receptor in cerebral ischemia may be critical for identifying the specific role of the GluN2A subunit, which may mediate pro-survival effects following cerebral ischemia.

## Expression of the GluN1/2A/2B receptor in the rat adult forebrain

The results of a sequential immunoprecipitation study revealed that 60–70% of GluN2A and 70–85% of GluN2B subunits in the CA1/CA2 of rat hippocampus are diheteromeric (Al-Hallaq et al., 2007). However, other studies suggest that the majority of NMDARs in the adult forebrain are triheteromeric GluN1/2A/2B receptors. Using quantitative immunoprecipitation analysis, Luo et al. found that the dominant NMDAR complex in the adult rat cortex contains three subunits, i.e., GluN1, GluN2A, and GluN2B (Luo et al., 1997). Subsequently, in a comparison of the NMDAR-mediated excitatory postsynaptic current decay time of wild-type mice with those of Grin2A^−/−^ and Grin2B^ΔFb^ mice, Rauner et al. demonstrated that the triheteromeric GluN1/2A/2B receptor is prominently expressed in the CA1 synapses of adult wild-type mice (Rauner and Köhr, 2011). Tovar et al. reported that at least two-thirds of NMDARs in hippocampal synapses are triheteromeric receptors, based on an analysis of the NMDAR deactivation kinetics in response to the competitive GluN2A antagonist NVP-AAM077 (Tovar et al., 2013). Delaney et al. found that the triheteromeric GluN1/2A/2B receptor is the primary synaptic NMDAR in principal neurons of the basolateral amygdala (Delaney et al., 2013). Through examining the relationship between spatial cognition and protein–protein interactions of GluN2B-containing NMDARs, Zamzow et al. speculated that there were more GluN1/2A/2B receptors in older mice than in young adults (Zamzow et al., 2013). Together, these data indicate that the triheteromeric GluN1/2A/2B receptor is the most common NMDAR in the adult forebrain.

## Pharmacological properties of the GluN1/2A/2B receptor

Recently, two independent research groups specifically expressed the triheteromeric GluN1/2A/2B receptor on the cell surface using the trafficking control system of GABA_B_ receptors, while the cell surface expression of diheteromeric NMDARs (GluN1/2A and GluN1/2B receptors) was inhibited (Hansen et al., 2014; Stroebel et al., 2014). Several lines of evidence show that compared to the selective GluN2B antagonists, selective GluN2A antagonists profoundly inhibit the GluN1/2A/2B receptors. The IC_50_ of TCN-201, a selective GluN2A antagonist, increased from 370 ± 30 nM for the wild-type GluN1/2A receptor to 1350 ± 130 nM for the GluN1/2A/2B receptors, and maximal inhibition was reduced from 91 ± 1 to 72 ± 4% (Hansen et al., 2014). Compared to that of the wild-type GluN1/2B receptor, the IC_50_ of ifenprodil, a selective GluN2B antagonist, increased 6.3-fold for the triheteromeric GluN1/2A/2B receptor, and maximal inhibition was reduced to 32 ± 1% (Hansen et al., 2014). Consistent with these results, Cheriyan et al. found that the GluN2A-selective inhibitors TCN-201 and NVP showed a similar inhibitory effect on diheteromeric and triheteromeric GluN2A-containing receptors, while the GluN2B-selective inhibitors ifenprodil, con-G, and con-RlB only inhibited triheteromeric GluN1/2A/2B receptor currents by 19.14, 5.5, and 14.3%, respectively (Cheriyan et al., 2016). Interestingly, Stroebel et al. observed a biphasic curve when evaluating the ifenprodil-induced inhibition of the GluN1/2A/2B receptor, and the majority of this inhibition, which increased 77%, was due to the low-affinity component (Stroebel et al., 2014). Thus, the GluN1/2A/2B receptor is sensitive to selective GluN2A antagonists. As a result, the effects of GluN2A antagonists may be due to the blockage of both GluN1/2A and GluN1/2A/2B receptors, which may confound investigations on the role of the GluN2A subunit.

## The role of the GluN1/2A/2B receptor in cerebral ischemia

**Figure 1:**
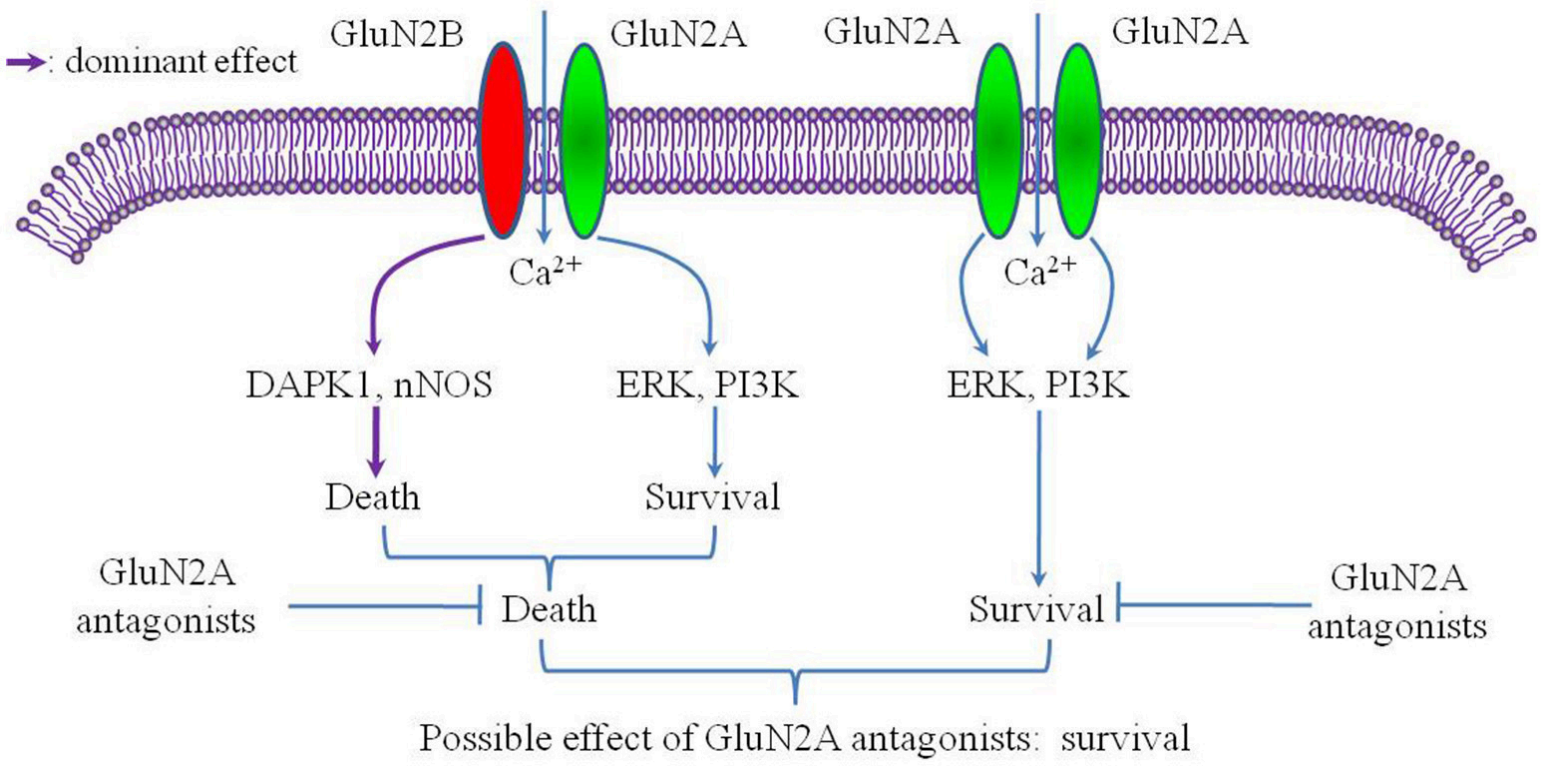
**Putative mechanism of selective GluN2A antagonism after cerebral ischemia**. The conditions of this putative mechanism include the GluN2A subunit mainly mediating a pro-survival effect after cerebral ischemia and the GluN1/2A/2B receptor acting as a pro-death factor with a more dominant role than the GluN1/2A receptor.

GluN2B promotes cell death following cerebral ischemia, activating many pro-death signaling molecules, such as neuronal nitric oxide synthase (nNOS) and death-associated protein kinase 1 (DAPK1) (Lai et al., 2014). However, the role of GluN2A in cerebral ischemia remains controversial (Sun et al., 2016). Notably, previous studies of cerebral ischemia have ignored the influence of the triheteromeric GluN1/2A/2B receptor despite the fact that this receptor accounts for a large proportion of the NMDARs in the adult forebrain. Considering the strong inhibitory effect of GluN2A-specific antagonists on the triheteromeric GluN1/2A/2B receptor, the role of the GluN2A subunit in cerebral ischemia may be worth further consideration. For example, although the GluN1/2A receptor might mediate pro-survival signaling during cerebral ischemia, the GluN1/2A/2B receptor could either promote cell survival or promote cell death. If the GluN1/2A/2B receptor promotes cell death and plays a more dominant role than the GluN1/2A receptor, the total effect of GluN2A antagonists on a cell expressing both the GluN1/2A/2B receptor and the GluN1/2A receptor might be pro-survival (Figure 1). In this circumstance, the pro-survival effect of GluN2A antagonists would incorrectly suggest that the GluN2A subunit promotes cell death during cerebral ischemia, leading to a misunderstanding of the role of GluN2A in cerebral ischemia. Given this possibility, further studies should investigate the effect of the triheteromeric GluN1/2A/2B receptor on cerebral ischemia. Papouin et al. found that the preferential GluN2A antagonist zinc, but not the selective GluN2B antagonist Ro25-6981, had a significant neuroprotective effect on the neuronal death induced by NMDA exposure in acute hippocampal slices (Papouin et al., 2012). Given the high expression level of the GluN1/2A/2B receptor in the hippocampal neurons (Rauner and Köhr, 2011) and the significant inhibitory effect of zinc on the GluN1/2A/2B receptor (Hansen et al., 2014), the triheteromeric NMDARs, but not the GluN1/2A receptor, located at synaptic sites may mediate the neurotoxic effect of NMDA on CA1 pyramidal neurons. Such a mechanism is consistent with our hypothesis.

## Perspectives

Investigating the GluN1/2A/2B receptor may lead to a re-evaluation of the treatment strategies for several neurological diseases related to excitotoxicity, such as stroke, Alzheimer's disease (AD), and Huntington's disease (HD). Previous studies suggest that for AD or HD, similar to stroke, overactivation of the NMDAR results in neuronal damage (Dau et al., 2014; Guivernau et al., 2016). However, it is still unclear whether the GluN1/2A or GluN1/2A/2B receptor mediates amyloid-beta- or mutant huntingtin-induced neurotoxicity. The GluN1/2A receptor and the GluN1/2A/2B receptor may play different roles in these diseases. Further studies should investigate whether blockage of the GluN1/2A/2B receptor protects against stroke, AD, or HD. If so, the GluN1/2B and GluN1/2A/2B receptor antagonists might have a better application prospect than existing GluN2B antagonists. Research in this area may also contribute to investigating whether the failure of GluN2B antagonists in clinical trials was due to the poor selectivity of GluN2B antagonists on the GluN1/2A/2B receptor.

NMDAR hypofunction may play a critical role in the pathophysiological process of schizophrenia, and NMDAR agonist treatment may improve patient symptoms (Kantrowitz et al., 2016). In an effort to avoid excitotoxicity, we should clarify whether a selective GluN1/2A receptor agonist is more effective than a non-selective GluN1/2A and GluN1/2A/2B receptor agonist.

Some evidence suggests that overactivation of synaptic NMDARs is responsible for neurodegeneration (Papouin et al., 2012; Zhou et al., 2015). However, this idea is inconsistent with the current prevailing theory that describes extrasynaptic NMDARs as a major pro-death factor (Hardingham and Bading, 2010). Notably, previous studies have not taken into account the effect of the GluN1/2A/2B receptor. Understanding the role of the GluN1/2A/2B receptor may be the key to explaining these conflicting results.

Future studies should clarify the subcellular distribution, signaling, and physiological and pathological functions of the GluN1/2A/2B receptor. Such research will lead to insight on the physiological and pathological functions of the three NMDAR subtypes found in the adult forebrain, and the specific roles of the GluN2A and GluN2B subunits. Current NMDAR antagonists show varying degrees of specificity; thus, selective antagonists of the GluN1/2A, GluN1/2B, and GluN1/2A/2B receptor are urgently needed. GluN2A or GluN2B antisense oligonucleotides and peptide mimetics may also be useful in this regard.

## Author contributions

YS wrote the manuscript and drew the figure. LW wrote part of the manuscript. ZG wrote the manuscript and approved the final version.

## Funding

This work was supported by the Natural Science Foundation of China (NSFC 81200886, NSFC 81402886), the Natural Science Foundation of Hebei Province (H2014208004).

### Conflict of interest statement

The authors declare that the research was conducted in the absence of any commercial or financial relationships that could be construed as a potential conflict of interest.
